# When the heart becomes suicidal: a case report of severe left ventricular outflow tract obstruction following transcatheter aortic valve implantation

**DOI:** 10.1093/ehjcr/ytaf164

**Published:** 2025-05-07

**Authors:** Carl Schulz, Fabian J Brunner, Simon Pecha, Nils Sörensen, Niklas Schofer

**Affiliations:** Department of Cardiology, University Medical Center Hamburg-Eppendorf, Martinistreet 52, 20251 Hamburg, Germany; Department of Cardiology, University Medical Center Hamburg-Eppendorf, Martinistreet 52, 20251 Hamburg, Germany; Department for Cardiovascular Surgery, University Medical Center Hamburg-Eppendorf, Martinistreet 52, 20251 Hamburg, Germany; Department of Cardiology, University Medical Center Hamburg-Eppendorf, Martinistreet 52, 20251 Hamburg, Germany; Department of Cardiology, University Medical Center Hamburg-Eppendorf, Martinistreet 52, 20251 Hamburg, Germany

**Keywords:** Case report, TAVI-related complication, Suicide ventricle, SAM, TASH, LVOT obstruction

## Abstract

**Background:**

Replacement of the aortic valve is a Class I recommendation for treatment of patients suffering from severe, symptomatic aortic stenosis. However, aortic valve replacement can occasionally lead to complications, including development of acute left ventricular outflow tract (LVOT) obstruction. This rare but severe complication is referred to as the so-called ‘suicide left ventricle’ phenomenon.

**Case summary:**

This case report presents an 88-year-old woman who developed severe LVOT obstruction following a successful transcatheter aortic valve implantation (TAVI), complicated by septal anterior motion of the mitral valve resulting in severe mitral regurgitation. Despite initial intensive care management, her symptoms persisted, necessitating the application of transcoronary ablation of septal hypertrophy as a bail-out procedure. Transcoronary ablation of septal hypertrophy, typically used in hypertrophic obstructive cardiomyopathy, successfully reduced the LVOT gradient and relieved symptoms.

**Discussion:**

This case emphasizes the importance of pre-operative identification of LVOT obstruction risk factors, awareness for this complication and a well-experience multidisciplinary team for the management of TAVI-associated complications.

**Learning points**

- Suicide left ventricle is a serious complication after (transcatheter) aortic valve replacement that needs to be managed by experienced teams using a stepwise therapy algorithm
- Predictors need to be identified during transcatheter aortic valve implantation (TAVI) evaluation and awareness of this phenomenon needs to be raised in at-risk patients undergoing TAVI

## Introduction

Replacement of the aortic valve (AVR) is a Class I recommendation for treatment of patients suffering from severe, symptomatic aortic stenosis.^1^ A rare, but serious complication following AVR is left ventricular outflow tract (LVOT) obstruction. This case report delves into a complex case of transcatheter aortic valve implantation (TAVI)-induced LVOT obstruction, highlighting the complexity and importance of diagnosis and management of this complication. Additionally, we show the use of transcoronary ablation of septal hypertrophy (TASH), a technique traditionally employed in hypertrophic obstructive cardiomyopathy (HOCM) patients, as a bail-out procedure in this clinical scenario.

## Summary figure

**Figure:**
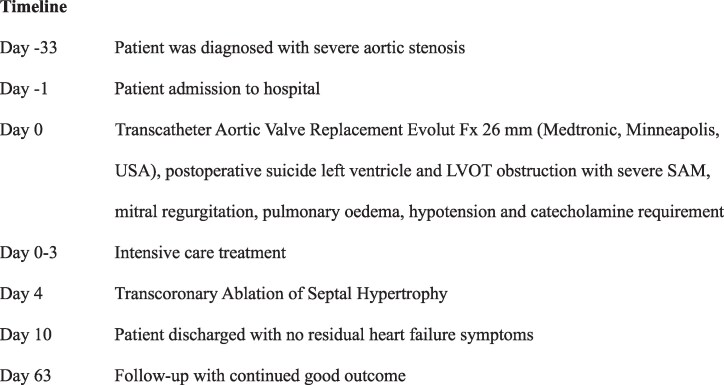


## Case presentation

**Figure 1:**
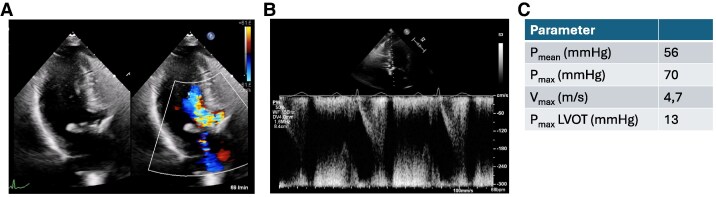
Transthoracic echocardiography before transcatheter aortic valve implantation treatment. (*A*) Apical three-chamber view of the aortic valve in systole showing severe flow acceleration and (*B*) pulse wave Doppler in the left ventricular outflow tract. (*C*) Doppler over the aortic valve.

**Figure 2:**
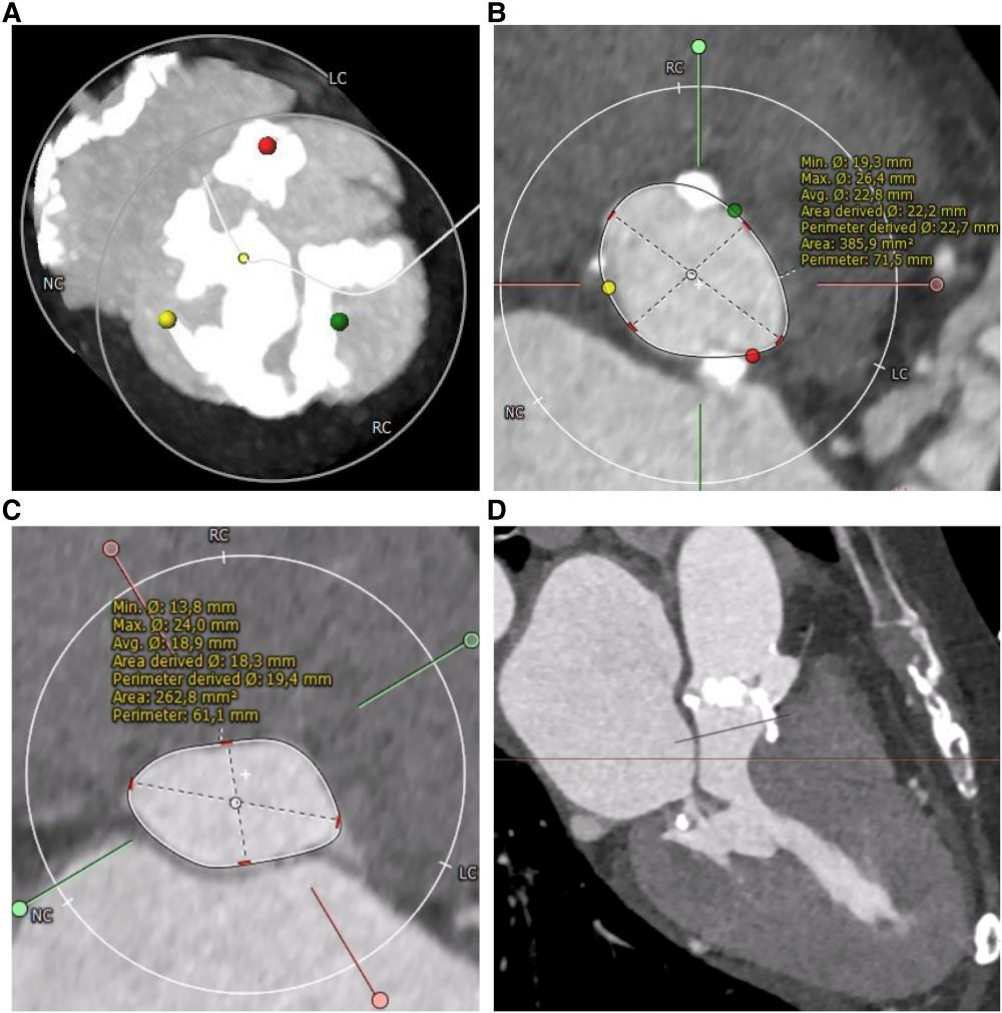
Pre-operative end-systolic computed tomography of the heart. (*A*) Computed tomography scan of the aortic valve shows severe calcification. (*B*) Annular assessment. (*C*) Assessment of the left ventricular outflow tract (LVOT) 8.5 mm below annular plane. (*D*) Left heart section showing left ventricular hypertrophy and small left ventricular chamber.

**Figure 3:**
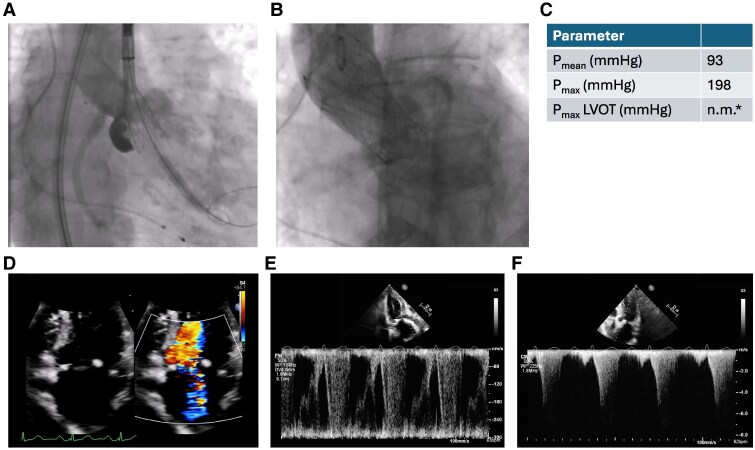
Transcatheter aortic valve implantation procedure and post-operative echocardiography. (*A*) Release of a self-expanding valve (Evolut Fx 26 mm, Medtronic, Minneapolis, MN, USA). (*B*) Transcatheter aortic valve implantation prosthesis after implantation without relevant aortic regurgitation. (*C*) Quantitative parameters in transthoracic echocardiography. *P_max_ in the left ventricular outflow tract was not measurable by PW-Doppler (see *P*_max_ by CW-Doppler). (*D*) Post-operative transthoracic echocardiography shows severe mitral regurgitation (*D*) and high left ventricular outflow tract gradients (*E* and *F*).

An 88-year-old woman presented with dyspnoea [New York Class Association (NYHA) III)] and was diagnosed with severe aortic stenosis. Clinical examination revealed a systolic murmur, vesicular breath sound, no peripheral oedema, and no clinical signs of hypoperfusion. The patient's medical history included subclinical hyperthyroidism and well-controlled arterial hypertension without other comorbidities [body mass index (BMI) 21.3 kg/m^2^, STS score 6.87]. Laboratory tests showed elevated NT-proBNP levels (3360 ng/L) without other relevant changes. Transthoracic echocardiography showed severe aortic stenosis (*V*_max_ 4,7 m/s, *P*_mean_ 52 mmHg, *P*_max_ 70 mmHg, left ventricular ejection fraction 55%, *Figure 1*) and severe left ventricular hypertrophy with mild LVOT obstruction. Computer tomography (CT) showed severe calcification of the aortic valve (*Figure 2*). Interdisciplinary case evaluation by the structural heart team led to the decision against operative aortic valve replacement with myectomy and to proceed with transfemoral TAVI for the treatment of aortic stenosis, taking into account the patient's request and advanced age (STS score 6.87, EuroSCORE II 7.8%). A self-expanding Evolut Fx 26 mm (Medtronic, Minneapolis, MN, USA) valve was implanted in adequate position under fast ventricular pacing without regurgitation according to aortic root angiography (*Figure 3A* and *B*).

**Figure 4:**
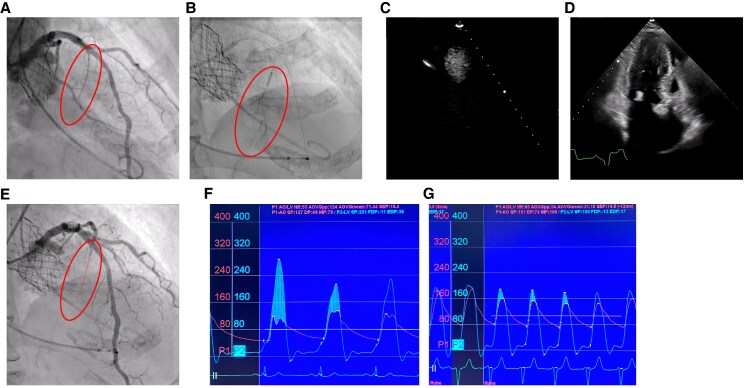
Transcoronary ablation of septal hypertrophy after transcatheter aortic valve implantation as a bail-out procedure. (*A*) Invasive coronary angiography showing the large S1 branch as the transcoronary ablation of septal hypertrophy target. (*B*) Angiography through the over-the-wire balloon showing contrast agent only distal to the balloon and not proximal, validating the proximal occlusion of the S1. (*C* and *D*) Periprocedural transthoracic echocardiography shows SonoVue in the septum for the target zone validation. (*E*) Coronary angiography after transcoronary ablation of septal hypertrophy shows total occlusion of the S1 and TIMI III flow in the left coronary artery. Invasive pressure gradient estimation before (*F*) and after (*G*) transcoronary ablation of septal hypertrophy.

**Figure 5:**
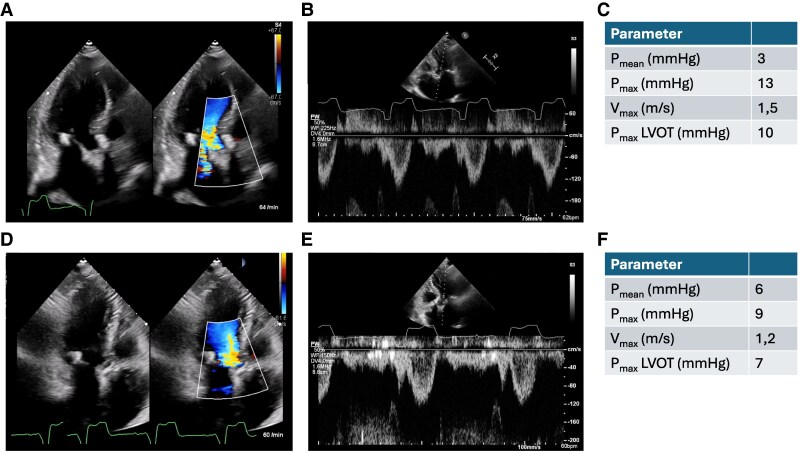
Apical three-chamber view, pulse wave Doppler in the left ventricular outflow tract and Doppler over the aortic valve 10 days after transcatheter aortic valve implantation (*A–C*) and 9 weeks after transcatheter aortic valve implantation (*D–F*).

Immediate after the procedure the patient presented with increasing respiratory insufficiency due to pulmonary oedema, hypotension, and increasing catecholamine requirement. Bedside transthoracic echocardiography showed high LVOT gradients (*Figure 3C*) and septal anterior motion (SAM) of the anterior mitral valve leaflet resulting in severe mitral regurgitation. Volume substitution and over-pacing with a temporary transjugular pacemaker failed to improve cardiac output. By applying non-invasive ventilation and administration of norepinephrine (up to 3 µg/min), the patient could be stabilized and was transferred to the intensive care unit (ICU). After 3 days in ICU, the patient was transferred to the regular unit; however, still oxygen dependent and being highly symptomatic (NYHA III and IV). Transthoracic echocardiography on Day 3 showed persistent, severe LVOT obstruction (LVOT gradient: *P*_max_ 198 mmHg, *P*_mean_ 93 mmHg), ‘kissing ventricle’ and SAM resulting in a significant increase in the mitral regurgitant jet (*Figure 3D–F*). The implanted TAVI prosthesis showed a good functional outcome. Further therapeutic options were discussed in the interdisciplinary structural heart team and the HOCM board. Due to the patient's persistent severe clinical symptoms and lack of prospect of spontaneous clinical improvement, a decision was made to proceed with TASH as a bail-out strategy. Peak-to-peak gradient across the LVOT was 80–100 mmHg by means of invasive measurement. Coronary angiography showed a large first septal branch (S1) as a suitable target for TASH (*Figure 4A*). A 6-Fr judkins guide catheter in combination with a guide extension catheter was used to engage the left coronary artery. A coronary wire was navigated into the distal part of left anterior descending (LAD) and a 2 mm × 8 mm over-the-wire (OTW) balloon was advanced over a coronary wire (*Figure 4B*) into the S1. While occluding, the S1 SonoVue (Bracco, Milan, Italy) was applied through the OTW balloon catheter validating an adequate target within the ventricular septum for TASH according to echocardiography (*Figure 4C–E*). After placing a transjugular pacemaker in the right ventricular (RV), 95% ethanol was applied incrementally (total 1.8 mL) via the OTW balloon catheter. During TASH, the peak-to-peak gradient across the LVOT decreased continuously (*Figure 4F* and *G*). After full-dose ethanol application persistent complete atrioventricular block occurred and the transjugular pacemaker was used to provide ventricular stimulation. At the end of the procedure, the LVOT peak-to-peak gradient had decreased to 30 mmHg. Final angiography of the left coronary artery showed TIMI III flow in the LAD and a proximal occlusion of the S1 (*Figure 4E*), the patient underwent implantation of a permanent pacemaker the same day. Clinical evaluation showed improvement in dyspnoea and exercise capacity. Echocardiography on Day 10 showed significant improvement with lower LVOT gradients (*P*_mean_ 3 mmHg and *P*_max_ 10 mmHg) moderate mitral regurgitation and mild SAM (*Figure 5A–C*). The patient was discharged on Day 11 on bisoprolol (7.5 mg per day), sodium perchlorate (1200 mg per day), and acetylsalicylic acid (100 mg per day) without residual heart failure symptoms. Follow-up 9 weeks after discharge showed a sustained good outcome with no relevant clinical symptoms (NYHA I), good function of the TAVI prosthesis (*P*_max_ 9 mmHg, *P*_mean_ 6 mmHg) without relevant LVOT gradients or mitral regurgitation (*Figure 5D–F*).

## Discussion

The presented case demonstrates severe LVOT obstruction complicated by severe SAM-induced mitral regurgitation as a possible adverse event that can occur immediately after aortic valve replacement, in this case TAVI. This so-called suicide left ventricle phenomenon is a rather rare complication after TAVI occurring in only 4–5% of cases.^2^ Mild LVOT obstruction after TAVI can usually be managed medically with a good prognosis.^2^ However, severe AVR-related LVOT obstruction is associated with a high risk of acute heart failure and short-term mortality. In those cases, advanced therapies may be required.^3^ Predictors of LVOT obstruction after TAVI are easily assessed by transthoracic echocardiography (asymmetric LV hypertrophy, small LV, high ejection fraction, SAM, high LVOT pressure gradients, Barzallo *et al*.^4^), and CT scan (subaortic septal bulge, small LV). Pre-operative identification of patients at risk allows planning of individualized treatment strategies and creates awareness for appropriate management of post-operative complications and clinical deterioration. Although some anatomical predictors for potential suicide left ventricle after TAVI were present in the presented case, such as severe LV hypertrophy and mild SAM, pre-emptive TASH^5^ was considered not to be justified as the critical, highly calcified aortic valve stenosis was identified as the main cause for outflow obstruction and symptoms. Therefore, we proceeded with TAVI with the awareness of acute post-procedural worsening of LVOT obstruction. A pre-procedural assessment of the anatomical suitability of TASH as a potential bail-out treatment was performed. However, clinical decision making in such borderline cases is difficult and TASH prior to TAVI may also be an option especially if there are echocardiographic signs of severe LVOT flow acceleration. Treatment of LVOT obstruction after TAVI is challenging and should follow a standardized and staged treatment algorithm. The first steps of medical management should include increasing preload (normovolaemia), increasing afterload (vasoconstrictors), decreasing inotropy (no inotropics, applying beta blocker), RV pacing to increase RV/LV dyssynchrony, followed by invasive bail-out strategies such as TASH. Veno-arterial extracorporeal membrane oxygenation (ECMO) may be required as a bridging therapy in patients with persistent haemodynamic instability.^6,3,7–10^ Evaluation of each step and treatment should be performed by a multidisciplinary and experienced team. Pre-operative identification of patients at risk is important and high awareness of the occurrence of AVR-associated suicide left ventricle is needed for appropriate response and therapy. The level of evidence for TASH after AVR-associated LVOT obstruction is rather low, as there are only anecdotal reports and no systematic studies of these patients. Other conservative treatment options such as novel myosin inhibitors (mavacamten) to reduce septal hypertrophy may be considered in stable patients in order to improve longer-term outcome.^11^

## Conclusion

Suicide left ventricle is a serious complication after TAVI that needs to be managed by experienced teams using a stepwise therapy algorithm. Predictors need to be identified during TAVI evaluation and awareness of this phenomenon needs to be raised in at-risk patients undergoing TAVI. Most importantly, expertise in advanced bail-out strategies such as TASH or vaECMO should be provided, when treating patients at risk for this rare, but serious complication.

## Supplementary Material

ytaf164_Supplementary_Data

## Data Availability

The data underlying this article are available in the article and in its online Supplementary material.

## Lead author biography

Dr. med. Carl Schulz is a resident in the Department of Cardiology at the University Medical Center Hamburg-Eppendorf. His main academic and clinical interests focus on structural heart diseases, aiming to contribute to advancements in the understanding and treatment of valvular disorders.

## Notes

**Consent:** Written informed consent was obtained from the patient for the publication of this case report, including all associated images and clinical details. The patient was informed that their identity would remain confidential and that no identifying information would be included in the publication. A copy of the signed consent form is available for review by the journal's editorial team upon request.

**Funding:** None declared.
